# Giving voice to those directly affected by the COVID-19 pandemic – the experience and reflections of a person with dementia

**DOI:** 10.12688/hrbopenres.13063.2

**Published:** 2020-11-05

**Authors:** Helen Rochford-Brennan, Fiona Keogh

**Affiliations:** 1 Irish Dementia Working Group, Alzheimer Society of Ireland, Dublin, Ireland; 2 Centre for Economic and Social Research on Dementia, National University of Ireland, Galway, Galway, Ireland

**Keywords:** Dementia, Covid19, PPI, experience

## Abstract

The coronavirus disease 2019 (COVID-19) pandemic presents unprecedented challenges to society. Behind the daily tally of deaths and cases of infection are individuals and families who are experiencing the ultimate consequence of this disease. Every aspect of our lives has been affected and these affects are amplified for those who have to cocoon and have conditions such as dementia. There is little opportunity to directly hear the experience of those ‘vulnerable adults’ who have been self-isolating for many weeks now.  This letter takes the form of a reflective conversation with a person living with dementia. Honouring the principles of public and patient involvement (PPI), it is an attempt to give voice to the experience of one of the many thousands of vulnerable people during the COVID-19 pandemic. As well as describing the effect on her daily life, Helen describes what supports would help at this time.  While the focus of attention at the moment is rightly on dealing with the effects of the virus in nursing homes, the many thousands of people living with dementia in the community should not be forgotten.

## Disclaimer

The views expressed in this article are those of the author(s). Publication in HRB Open Research does not imply endorsement by the Health Research Board of Ireland.

## 

This idea for this letter was inspired by an initial conversation with Helen Rochford Brennan (HR-B) who has been cocooning for over seven weeks at time of writing. She and her husband both fit the criteria of ‘extremely medically vulnerable’ as both are over 70 and each has a medical condition; dementia and a heart condition respectively ( HPSC, 2020). Over the last five weeks Fiona Keogh (FK) from the Centre for Economic and Social Research on Dementia at NUI Galway had a series of follow-up conversations with HR-B about her experience of the coronavirus disease 2019 (COVID-19) emergency measures, how her life is affected and supports she has received from the health and social care system.

As the pandemic emerged, the emphasis was rightly on emergency measures, clinical care, hospital readiness and rapid responses. The voice of experts in epidemiology, infectious disease and public health necessarily dominated the information flows. These voices have provided welcome clarity and reassurance in difficult times. Their guidance has been widely respected and closely followed in Ireland and most other countries. As we move into the third month of COVID-19 in Ireland and the easing of some restrictions, we can begin the process of reflecting on the experience thus far, what has been learned and what our priorities might be in the coming months. This letter aims to provide a contemporaneous account of the experience of a person with dementia of living through this pandemic in order to inform these considerations. The practice of public and patient involvement (PPI) in research has become mainstreamed to the extent that many research funders require evidence of PPI in funding applications (for example, the Health Research Board). PPI recognises the unique value of the personal experience in understanding a medical condition and bringing that experience to bear in research and policy development ( Ocloo & Matthews, 2016; Sabat, 2003). The right to be heard and to be involved in research and policy is particularly important for people who are marginalised or whose condition makes it less likely that their voice will be heard ( Cahill, 2019; Swaffer, 2018). Dementia is one such condition. People with dementia are mostly older; placing them at high risk of COVID-19. As dementia progresses it can affect a person’s ability to communicate or to comprehend complex information, such as that provided during a pandemic. They may be judged as having ‘no capacity’, thereby taking away their right to make almost any decision. If PPI was important before the pandemic, it is even more important now, to ensure that the voice of vulnerable people is heard at all stages of dealing with the current crisis. Those who will be directly impacted must have a role in shaping what health and social care services will look like now and into the future, and this is particularly true for people with dementia.

Helen lives at home with her husband in the outskirts of a small town in the west of Ireland. Their son lives and works in London. She was diagnosed with early onset Alzheimer’s disease in 2012 at the age of 62. Up to the time of diagnosis, Helen had a busy working life. Some time after her diagnosis, she became an active campaigner for rights and social justice in dementia. Her advocacy has led to her becoming involved in research on dementia which provided her with “a great outlet as well as an opportunity to meet and get support from peers”. She became an active member of the Irish Dementia Working Group (IDWG) which led to her involvement with Alzheimer Europe and the European Working Group of People with Dementia (EWGPWD). She was elected Chairperson of this group in 2016 and serves on the Board of Alzheimer Europe in this capacity. Helen acknowledges that she may not seem like a ‘typical’ person with dementia but she reminds me that everyone has a life before dementia and that many aspects of her life are similar to many others; “every person’s circumstances are different and we each experience dementia in different ways so what is typical anyway?” While this letter describes Helen’s own experience, through her advocacy work and contact with peers, she is also drawing on the experience of others in her account.

Asked to describe the effect of cocooning on her life, Helen describes heightened anxiety, stress and isolation. The anxiety is ever-present – the ‘what if’ scenarios that play out in her head continuously. As well as having dementia, Helen is the carer for her husband and is finding this responsibility particularly stressful at this time. Normally if she felt anxious or stressed Helen would go for a walk or meet friends for a chat. These outlets are not available now. This sense of being cut-off and isolated, adds to the anxiety and stress. In this time period, Helen became physically unwell herself, emphasising the dynamic nature of everyday life and the need to keep modifying supports and responses as a person’s circumstances change. These challenges are similar to those of the many older people and people with medical conditions who are cocooning ( Alzheimer Society of Ireland, 2020). However, dementia brings additional challenges. Forgetfulness creates obvious difficulties. People with dementia may need reminding to implement the basic guidance around social distancing. Small everyday occurrences for many of us are magnified for Helen, such as misplacing a small medical device which cannot easily be replaced but is needed every day.

A critical challenge at this time is that people with dementia often don’t seek out help because they may not realise they need it or they can forget what help is available, particularly if it is outside the usual range of supports ( Werner *et al*., 2014). For example, the Gardai and the postal employees are providing extra support at this time, but Helen forgot about this until a Garda called to her house to enquire if she needed anything. Another concern that is particularly relevant for people with dementia is ‘use it or lose it’ - the acceleration in loss of abilities and cognitive function in the absence of the stimulation posed by everyday living situations such as shopping, using transport, social situations and so on ( Aguirre *et al.,* 2013; Mitchell, 2018). Helen articulates this concern strongly and describes her strategies to overcome it such as telephone conversations, attending virtual meetings and baking, which provides the discipline of following a recipe and remembering each step.

Helen and her husband receive 30 minutes of home support each week. Her husband receives support for his heart condition from a day hospital, with check-in phone calls and attendance at the day hospital if necessary. Helen also spent a short time in hospital and received follow-up care. Apart from these formal services, the only other formal support available to Helen is information and online resources. Helen called some helplines to assess what support might be available for people with dementia. These directed her to local services – none of which were operating at this time. Since this letter was first written in April/May 2020, additional support has come on stream for Helen. She now has zoom contacts with a dementia nurse specialist using her laptop. She also had a virtual consultation with a doctor from the dementia services team using the virtual assistant assistive technology device ‘Alexa’. She is also receiving support for her role in caring for her husband. These contacts emphasise the usefulness and importance of technology in enabling new forms of service delivery.

Helen welcomes the *Community Call* initiative ( Department of Rural and Community Development, 2020), and this approach of mobilising community support is very much in line with what she has advocated for many years. While she can access online information and seek support from *Community Call* fairly easily, Helen is conscious of the challenges for people with dementia. Firstly, the person must be able to identify and articulate their support needs, and secondly, they need to *proactively* seek out the relevant information, which is mostly online. The person also needs the necessary equipment (computer or smartphone), infrastructure (broadband connection) and skills to navigate online. Helen emphasises two practical supports which would really help people with dementia at this time. Firstly, human contact which is essential for reassurance and emotional support; and secondly, a simple information sheet specifically for people with dementia and their carers, sharing strategies and information they need at this time.

In spite of the fact that almost two thirds of people with dementia in Ireland live in the community, people with dementia can be ‘invisible’ in many respects ( Pierse *et al.*, 2019). Many people with dementia are not diagnosed and are therefore are not in contact with any health service for their dementia. It is estimated that the majority of community-dwelling people with dementia in Ireland do not have a diagnosis ( Cahill *et al.,* 2012). Many people who have a diagnosis use a wide range of generic (e.g. home support) and dementia-specific services (e.g. dementia adviser) with no centralised list. Practice-based registries could play a key role in identifying people with dementia who could be proactively contacted to establish their support needs and provide human contact and emotional support. The Chronic Disease Management Programme ( HSE, 2019) creates in effect, a practice-based register of people with specific conditions which could be used to proactively provide telephone support and advice to patients at this time. However, dementia is not part of this programme.

Helen names the many positives in her life – she is not living alone, she can get outside in her garden and she has a supportive network of family and neighbours. She is adept at computers and social media with access to good broadband. She has a wide network of peers and colleagues she can contact by phone. She uses these resources to keep herself active and to keep the isolation and anxiety at bay. She describes herself as resilient but observes that “resilience can only stretch so far”. Helen describes her own resilience as coming from her upbringing and her work and life experiences, but also from interventions that she has had the opportunity to access such as cognitive rehabilitation therapy (CRT). The latter is a psychosocial intervention which focuses on improving cognitive functioning in everyday life and supporting people to achieve the everyday goals that matter to them through developing and implementing strategies that work for them ( Bahar-Fuchs *et al.,* 2013). There is some evidence that people with dementia and their carers who have experienced CRT sustain what they learned and continue to develop strategies for the challenges posed by dementia ( Clare *et al.*, 2019).

Reflecting on Helen’s experience, we identified the importance of an existing service infrastructure which can be adapted to respond in times of crisis. However, community-based services for people with dementia have historically been fragmented, underdeveloped and underfunded compared to other services ( O’Shea *et al.,* 2017). There have been welcome developments in dementia services following on the publication of the National Dementia Strategy ( Department of Health, 2014) such as the implementation of psychosocial interventions. However, this was from a base of close to zero, and so CRT and other psychosocial interventions are only available on a very limited scale in Ireland. The poor availability of many dementia-specific services and the variety of services people may access, makes it particularly difficult to proactively reach out directly to people with dementia to provide support, which is exactly the kind of action needed at this time. This situation is likely exacerbated now by the need to direct scarce resources into nursing homes where the spread of the COVID-19 virus is most acute ( Department of Health, 2020). The years of neglect of community-based supports for people with dementia are more obvious now than ever. Even in the middle of the current crisis, this seems an inescapable fact that must be addressed at the first opportunity.

While formal health services are important, people with dementia have long recognised the importance of a social model of support ( Mental Health Foundation, 2015) and the development of dementia friendly or dementia inclusive communities has played an important role in building community responses, particularly in Ireland ( Alzheimer Society of Ireland, 2016). Initiatives such as the *Community Call* are building on decades of rural and urban community development programmes in Ireland, as well as more recent initiatives such Age Friendly Ireland and Dementia Inclusive Communities ( Age Friendly Ireland, 2020; HSE, 2020). That is not enough, however, and there will be plenty of lessons to be learned from the current crisis, not least the need to have significant investment in home care services and supports ( O’Shea, 2020a; O’Shea, 2020b). Keeping people out of nursing homes and building an eclectic continuum of care ( O’Shea *et al.,* 2019) should be the legacy of this terrible virus, according to Helen. We suggest the following should feature in this continuum:

- The continuation of contact through phone calls and practical support for virtual contact through increased availability of assistive technologies;
- Continuation of the *Community Call* initiative (or similar) to mobilise community support;
- A simple information sheet as well as human contact;
- The inclusion of people with dementia on the chronic disease management programme registers;
- Support for increased use of peer to peer support, for example, through a post-diagnostic course or similar shared learning forum.

The National Dementia Office and the HSE have been making great strides to effect change in the dementia landscape in Ireland. But to do the job properly, they need resources to fund the changes that are necessary to keep more people with dementia living well in their own homes.

## Consent

Written informed consent for publication of the participants’ details was obtained from the participants.

## Data availability

### Underlying data

No data are associated with this article

## References

[ref-1] Age Friendly Ireland: Age Friendly Ireland Programme. 2020 accessed 11/05/2020. Reference Source

[ref-2] Aguirre E Woods RT Spector A: Cognitive stimulation for dementia: A systematic review of the evidence of effectiveness from randomised controlled trials. *Ageing Res Rev.* 2013;12(1):253–262. 10.1016/j.arr.2012.07.001 22889599

[ref-3] Alzheimer Society of Ireland: Covid-19: Impact & Need for People with Dementia and Family Carers. 2020 Reference Source

[ref-4] Alzheimer Society of Ireland: Dementia Friendly Communities Toolkit. 2016 Reference Source

[ref-5] Bahar-Fuchs A Clare L Woods B: Cognitive training and cognitive rehabilitation for persons with mild to moderate dementia of the Alzheimer's or vascular type: a review. *Alzheimers Res Ther.* 2013;5(4):35. 10.1186/alzrt189 23924584 PMC3979126

[ref-6] Cahill S: Dementia and human rights.Bristol, Policy Press and Bristol University Press. 2019 Reference Source

[ref-7] Cahill S O’Shea E Pierce M: Creating excellence in dementia care: A research review for Ireland’s National Dementia Strategy.Dublin and Galway, Ireland: Trinity College Dublin and National University of Ireland, Galway. 2012 Reference Source

[ref-8] Clare L Kudlicka A Oyebode J: Individual goal-oriented cognitive rehabilitation to improve everyday functioning for people with early-stage dementia: A multicentre randomised controlled trial (the GREAT trial). *Int J Geriatr Psychiatry.* 2019;34(5):709–721. 10.1002/gps.5076 30724405 PMC6593854

[ref-9] Department of Health: National Dementia Strategy.Government of Ireland, Dublin. 2014 Reference Source

[ref-10] Department of Health: Statement from the National Public Health Emergency Team - Tuesday 14 April 2020. 2020 Reference Source

[ref-11] Department of Rural and Community Development: The Community Call. 2nd April 2020.2020 Reference Source

[ref-12] Health Protection Surveillance Centre: Guidance for vulnerable groups. 2020 accessed 11/05/2020. Reference Source

[ref-14] Health Service Executive: Chronic Disease Management Programme. 2019 Reference Source

[ref-15] Health Service Executive: Understand Together. 2020 Reference Source

[ref-21] Mental Health Foundation: Dementia, Rights and the Social Model of Disability. 2015 Reference Source

[ref-20] Mitchell W: Somebody I Used to Know. Bloomsbury Publishing. 2018 Reference Source

[ref-16] Ocloo J Matthews R: From tokenism to empowerment: progressing patient and public involvement in healthcare improvement. *BMJ Qual Saf.* 2016;25(8):626–32. 10.1136/bmjqs-2015-004839 26993640 PMC4975844

[ref-17] O’Shea D: The Dawning of a New Era for Health Care of Older People in Ireland?Irish Gerontological Society. 2020a Reference Source

[ref-19] O'Shea E: Remembering people with dementia during the COVID-19 crisis [version 1; peer review: 3 approved]. *HRB Open Res.* 2020b;3:15 10.12688/hrbopenres.13030.1 32510035 PMC7195897

[ref-18] O’Shea E Cahill S Pierce M: Developing and implementing dementia policy in Ireland.Galway. Centre for Economic and Social Research on Dementia. National University of Ireland Galway. 2017 Reference Source

[ref-23] O’Shea E Keogh F Cooney A: The Continuum of Care for People with dementia in Ireland.CESRD and the National Dementia Office. 2019 Reference Source

[ref-27] Pierse T O’Shea E Carney P: Estimates of the prevalence, incidence and severity of dementia in Ireland. *Irish J Psychol Med.* 2019;36(2):129–137. 10.1017/ipm.2018.31 31187725

[ref-24] Sabat S: Some Potential Benefits of Creating Research Partnerships with People with Alzheimer’s Disease. *Res Policy Plann.* 2003;21(2):5–12. Reference Source

[ref-25] Swaffer K: Human rights, disability and dementia. *Australian Journal of Dementia Care.* 2018;7:1 Reference Source

[ref-26] Werner P Goldstein D Karpas D: Help-Seeking for Dementia: A Systematic Review of the Literature. *Alzheimer Dis Assoc Disord.* 2014;28(4):299–310. 10.1097/WAD.0000000000000065 25321607

