# Supplementary material for: Giving voice to those directly affected by the COVID-19 pandemic – the experience and reflections of a person with dementia
Source: HRB Open Res. 2020 Nov 5;3:29. Originally published 2020 May 28. [Version 2] doi: 10.12688/hrbopenres.13063.2 (PMC7268151; doi:10.12688/hrbopenres.13063.2)
Supplement: Peer review history

# Peer review: Reviewer response for version 1

**DOI:** 10.21956/hrbopenres.14159.r27462

**Published:** 2020-06-03

**Authors:** Éidín Ní Shé

**Affiliations:** 1 School of Nursing, Midwifery and Health Systems, C129, UCD Health Sciences Centre, University College Dublin, Dublin, Ireland

It was a privilege to read this letter. The authors, using an inclusive and participatory approach, weave the lived experience of Rochford-Brennan within the literature and current policy and practice.

 The approach used in the writing of the letter is an exemplar of best practice in public and patient involvement I have come across.

 We are provided valuable insight into the daily life of Rochford-Brennan. She notes that cocooning has increased experiences of anxiety, stress and isolation as she navigates the pandemic and being the primary carer for her husband. The cracks in community supports are laid out by the authors that need urgent resources and integrated approaches.

 This letter should be read and reflected on by anyone interested in integrated patient-centred care in Ireland. Ideally, it should be at the top of the reading pile of our next government.

Does the article adequately reference differing views and opinions?

Yes

Are all factual statements correct, and are statements and arguments made adequately supported by citations?

Yes

Is the Open Letter written in accessible language?

Yes

Where applicable, are recommendations and next steps explained clearly for others to follow?

Yes

Is the rationale for the Open Letter provided in sufficient detail?

Yes

Reviewer Expertise:

Health Systems, Public and Patient Involvement, Co-Design.

I confirm that I have read this submission and believe that I have an appropriate level of expertise to confirm that it is of an acceptable scientific standard.

# Response

**Published:** 2020-10-12

**Authors:** Fiona Keogh

**Affiliations:** NUI Galway, Ireland

Many thanks Eidin for taking the time to review the letter and for your positive comments.

# Peer review: Reviewer response for version 1

**DOI:** 10.21956/hrbopenres.14159.r27463

**Published:** 2020-06-02

**Authors:** Damian Murphy

**Affiliations:** 1 Innovations in Dementia CIC, York, UK

This excellent Open Letter builds to a call for better and more sustainable community based support across Ireland for people living with dementia. If anything I feel the call could be more powerful and explicit. 

 This open letter reports on a series of conversations between the authors, Helen and Fiona. The conversations cover Helen's experiences and reflections as a person living with dementia in the community during the COVID-19 crisis in Ireland. With the current emphasis rightly being on the situation in long term care, the letter is an important reminder that the majority of people with dementia still live in the community and that this crisis has revealed some particular areas to address.

 The aim is for this contemporaneous account to help shape the priorities of health and social care services in the future. Helen's personal experiences and the reflections of both show how the COVID crisis has exacerbated issues of anxiety, isolation and the potential loss of confidence and skills amongst people living with dementia. Their conversation also highlighted some long standing gaps in community support that are in need of urgent filling.

**Rationale**

 I thought this was really clear. OK the focus is currently on long term care but we need to capture and understand the impact of this crisis with all groups - particularly those living with a dementia. Fiona rightly uses a Public Patient Involvement approach and highlights the need for individuals themselves to have a proper input in shaping future services coming out of this crisis.

 I would suggest that you could mention that the majority of people living with dementia in Ireland are in fact living in the community. (I'm guessing that is the case, knowing that in the UK it's approx 2/3 living in the community).

 Might you also include 'in the community' in the title?

**Range of views**

 It is difficult to reference a range of views when writing up an account of a personal lived experience. As far as the reflective element is concerned it is rightly important that Fiona and Helen addressed the possible criticism of Helen of not fitting the 'typical' idea of a person with dementia. 'What's typical anyway?' asks Helen. Her eloquence and remaining skills do not make her experience and comments any less valid. 

 It may be worth heading off any further critique of the letter involving a conversation with only one person with dementia by making it clear that Helen's positions on the European front are because she no doubt carries with her the stories and experiences of so many of her peers with dementia, living with a range of abilities across Ireland. Some of the reflections in the letter are clearly drawn from the experience of others.

 Further on in the letter, although they highlight a dearth of service provision, they rightly acknowledge community based initiatives that have worked or that still have potential, even if they are not so widely available across the country.

**Statements and arguments**

 In the 4th paragraph covering Helen's own challenges around anxiety and isolation, there is a throw away 'people...may need constant reminding' because of forgetfulness. There isn't a reference to this and although it may indeed be true I don't think it needs to be there, given the strength of the argument and key points to follow around rural and technological isolation. (Unless of course it was something you have both seen or had shared in your broader engagement with people with dementia).

 I think you are both right to correctly identify the threat of people with dementia being excluded and deprived of the opportunity to contribute to matters relevant to them. Would it be because of a blanket assumption of no capacity? I'm not aware of the law around capacity in Ireland. Are questions of capacity usually decision-specific? And if that is a basic right as you mention, where or from whom might this threat of exclusion come? It might be worth mentioning where and in what circumstances. (I'm thinking possibly close relatives, neighbours, local decision makers?)

**Language**

 'As dementia progresses it can rob the person of their ability to ...' It is possibly a personal thing but the term 'rob' always feels a little too loaded to me in a negative way, when we spend so much time promoting the ability and remaining potential of people not that we don't acknowledge the pain of things. May I suggest 'As dementia progresses it can affect a person's ability to...'. Otherwise I felt the whole letter to be very clearly written. Not always the case in many pieces of work!

**Recommendations and next steps**

 I really like what the open letter has done. This crisis has revealed a new marginalised group - those people with little or no access to technology and WiFi - as well as those existing marginalised people with dementia in the rural community. Many of these 'invisibles' fall in both groups, no doubt. Helen spoke of her own isolation too, giving top tips and flagging up some excellent initiatives.

 I think to the call for more funding of community services, Fiona and Helen could have been more explicit in laying out (in fact re-emphasising what they have already covered in the letter) exactly what an 'eclectic continuum of care' might look like.

- People not realising they need help and how to use it rather than lose it - calls and conversations, virtual meetings and the peer support that that brings.
- The community call initiative to mobilise community support.
- A strategy to provide WiFi access and the provision perhaps of 'stripped down' devices that can be easily used and accessed by people in remote areas.
- A simple information sheet together with a human contact.
- Practice-based registries - inclusion of people with dementia on the chronic disease management programme registers.

As a reader, the message of scant financial resources and extremely patchy provision of post diagnostic interventions such as CRT, got me thinking about exactly what practical suggestions other than more funding might work. Helen had already mentioned the benefit of virtual meetings ( I assumed with peers in the iDWG for example) so I drew from the letter this final recommendation:

- **much better use of the power of peer to peer support. **- This 'self management' approach was hinted at but not explicitly put forward and could come in the form of people with dementia using their own experience and skills to accompany others following diagnosis - either in a post diagnostic course or through shared learning and use of simple counselling type skills for example.

Does the article adequately reference differing views and opinions?

Yes

Are all factual statements correct, and are statements and arguments made adequately supported by citations?

Yes

Is the Open Letter written in accessible language?

Yes

Where applicable, are recommendations and next steps explained clearly for others to follow?

Partly

Is the rationale for the Open Letter provided in sufficient detail?

Yes

Reviewer Expertise:

Dementia and user involvement.

I confirm that I have read this submission and believe that I have an appropriate level of expertise to confirm that it is of an acceptable scientific standard.

# Response

**Published:** 2020-10-12

**Authors:** Fiona Keogh

**Affiliations:** NUI Galway, Ireland

Many thanks Damian for taking the time to review our letter. Your comments and suggestions are very helpful and we have addressed each of your comments and made changes to the letter as follows:

It is important to emphasise that most people with dementia in Ireland, as elsewhere, live in the community and we have included a reference to this at the beginning of paragraph 8.

Although Helen lives in the community, we decided not to amend the title in order to keep the scope of the letter inclusive. Some reports confirm that her experiences of anxiety and isolation are also relevant to people with dementia living in residential settings.

As  you note, although Helen is describing her own experience she is drawing on her conversations and interactions with other people with dementia before and during the pandemic. The text at the end of paragraph 3 has been included to reflect this.

In this dynamic environment, the supports to Helen have increased as services became more attuned to how they might operate as the pandemic continued. A brief description of these additional supports has been added to the end of paragraph 6.

**Statements and arguments**

The reference to the need for ‘continuous’ reminding because of forgetfulness has been dropped but we have kept the reference to the need for reminding in relation to social distancing as this is something Helen is concerned about herself.

The comment in the first paragraph was one of a series of reasons as to why we need hear the voice of people with dementia at this time. The law on capacity has changed in Ireland to protect decision making for those whose capacity may be affected, although it has not been fully implemented as yet. While a consideration of capacity is important, it is not an issue we explore in detail within the limited scope of a letter format.

**Language**

We agree it is important to emphasise the ability and remaining potential of people with dementia. The word ‘rob’ has been deleted.

**Recommendations and next steps**

A summary of the suggestions and recommendations is very helpful and we have included this at the end of the letter.

Your point about peer support is really important – it is one of the main areas of support for Helen and we did not make that explicit enough. We are happy to emphasise the importance and potential benefits of peer support.

# Peer review: Reviewer response for version 1

**DOI:** 10.21956/hrbopenres.14159.r27461

**Published:** 2020-06-01

**Authors:** Steven R. Sabat

**Affiliations:** 1 Department of Psychology, Georgetown University, Washington, DC, USA

This Open Letter, co-authored by a person living with dementia (HR-B) is important in shining necessary light on the situations faced by people living with dementia during the pandemic of the Coronavirus (COVID-19), the subjective experience of the lockdown, the needs that exist, and the support that would be helpful. 

 This focus is especially important because (1) great attention and resources have naturally been given to those ill with the virus, (2) there has been a focus on people living with dementia living in nursing homes, and (3) there was a need for resources and services, including diagnostic services, for people living in their own homes in Ireland before the pandemic that has been exacerbated ever since because of the need to "cocoon" which can exacerbate the social isolation and the accompanying stresses that are thus entailed. 

 Understanding the subjective experience of people diagnosed is of utmost importance for many reasons, not the least of which is the direction in which resources and services can be provided so as to help such people avoid having to live in nursing homes while maximizing the quality of their lives while living at home, where social and cognitive supports are extremely valuable. This Open Letter fulfills this need admirably.

 The references are all proper and cited appropriately. There are no "differing opinions" in this area of which I am aware. There is one typo in the Abstract, in the sentence that begins with "Every aspect of our lives..." The word, "affects" should be "effects".

Does the article adequately reference differing views and opinions?

Yes

Are all factual statements correct, and are statements and arguments made adequately supported by citations?

Yes

Is the Open Letter written in accessible language?

Yes

Where applicable, are recommendations and next steps explained clearly for others to follow?

Yes

Is the rationale for the Open Letter provided in sufficient detail?

Yes

Reviewer Expertise:

Alzheimer's disease and Dementia

I confirm that I have read this submission and believe that I have an appropriate level of expertise to confirm that it is of an acceptable scientific standard.

# Response

**Published:** 2020-10-12

**Authors:** Fiona Keogh

**Affiliations:** NUI Galway, Ireland

Thank you Steve for reviewing our open letter. Your comments have even more relevance as we begin to understand more about the impact of Covid-19 on people with dementia and other marginal groups.

The typo in the abstract has been corrected.
